# The Development and Validation of a Questionnaire That Assesses Female Athletes’ and Coaches’ Knowledge of the Menstrual Cycle

**DOI:** 10.1002/ejsc.70019

**Published:** 2025-08-23

**Authors:** Katharina Fischer, Elisabeth Maria Kirschbaum, Lucie Nikoleizig, Franziska Lautenbach, Kerry McGawley, Anne‐Marie Elbe

**Affiliations:** ^1^ Research Group Elite Female Athletes Institute for Applied Training Science Leipzig Germany; ^2^ Sports Psychology Faculty of Sports Science University of Leipzig Leipzig Germany; ^3^ Movement Biomechanics Institute of Sport Science Humboldt‐Universität zu Berlin Berlin Germany; ^4^ (Sports‐) Psychology Macromedia University of Applied Sciences Leipzig Germany; ^5^ Sports Psychology Institute of Sports Science Humboldt‐Universität zu Berlin Berlin Germany; ^6^ Swedish Winter Sports Research Centre Department of Health Sciences Mid Sweden University Östersund Sweden

**Keywords:** elite sports, instrument development, knowledge test, menstrual health, questionnaire construction

## Abstract

In this study, a menstrual cycle knowledge questionnaire (MCKQ) for female athletes and coaches was developed and validated. Items for an initial questionnaire (Pre‐MCKQ) were generated from existing questionnaires and supplemented by three experienced gynaecologists. The 21‐item Pre‐MCKQ was piloted on a sample of 325 recreational female athletes, 27 female coaches and 19 male coaches. Each item's difficulty and item–total correlation scores were assessed, and the final MCKQ was reduced to 12 items. Eighteen experts rated the 12‐item MCKQ for relevance (*M* = 5.4 and SD = 0.8) and clarity (*M* = 5.4, SD = 1.0) using a 6‐point Likert scale. The 12‐item MCKQ was then completed by 562 elite female athletes and 170 coaches (55 female, 114 male and 1 other). Test–retest reliability was assessed with a separate cohort of sport science students (*n* = 64). The mean difficulty index was 57%, and the mean item–total correlation was *r* = 0.36. Cronbach's alpha of 0.73 indicates that the questionnaire is reliable and test–retest reliability was strong. Knowledge test scores differed significantly among female athletes, female coaches and male coaches (*F* (2,728) = 8.59, *p* < 0.01 and *ηp* ^*2*^ = 0.02, respectively), demonstrating construct validity. Additionally, participants who rated their knowledge of the menstrual cycle as higher scored significantly better than those with lower self‐ratings (*F* (5,726) = 38.45, *p* < 0.01, *ηp* ^*2*^ = 0.21). In conclusion, the 12‐item MCKQ appears to be a valid and reliable tool to assess the menstrual cycle knowledge of female athletes and coaches.

## Key Points

- This study developed and validated a 12‐item menstrual cycle knowledge questionnaire (MCKQ) for female athletes and coaches, focusing on physiological menstrual cycle processes, menstrual irregularities and related symptoms.
- The MCKQ shows reliability (Cronbach’s alpha = 0.73), content validity (scale‐level content validity index = 97%), and construct validity, with significant differences in knowledge scores among female athletes, female coaches and male coaches, and between self‐rated knowledge levels. Test–retest reliability was also strong.
- Future research can use the MCKQ to evaluate the effectiveness of menstrual cycle educational programmes in sports contexts, enhancing menstrual health knowledge.

## 1 Introduction

The menstrual cycle (MC) is a biological process that women experience between puberty and menopause. Each cycle typically lasts 21–35 days, and the primary purpose is to prepare the female body for pregnancy. During the MC, concentrations of female‐dominant sex hormones (i.e., oestrogen and progesterone) fluctuate over the bleeding (menses), follicular, ovulatory and luteal phases (Janse De Jonge et al. 2019), and these hormone fluctuations influence a wide range of physical (e.g., cramps and fatigue), behavioural (e.g., comfort eating and mood swings) and psychological (e.g., anxiety, depression, and insomnia) symptoms.

Knowledge and communication about the MC are particularly relevant to female athletes and sports coaches (Laske et al. 2024). A scoping review of 39 studies by Oester et al. (2024) shows that between 2.8% and 100% of female athletes reported that their performance was adversely affected by their MC, the main reason being the occurrence of MC symptoms. This review highlights the high degree of variability in how athletes perceive themselves to be affected by their MC. Moreover, female athletes are more likely than the general female population to suffer from hormonal abnormalities due to an increased risk of low energy availability (i.e., a mismatch between dietary energy intake and energy expended during exercise, leaving the body’s total energy needs unmet; Mountjoy et al. 2023), which can lead to menstrual disturbances and disorders (Gimunová et al. 2022; Robert Koch‐Institut 2020; Taim et al. 2023). Menstrual disorders, such as amenorrhoea (i.e., the complete absence of menstrual bleeding), can impair sports performance and various physiological functions (e.g., energy metabolism, reproductive function, musculoskeletal health, immunity, glycogen synthesis, and cardiovascular and haematological health), which can individually and synergistically lead to impaired well‐being and increased injury risk (Mountjoy et al. 2014, 2023).

Female athletes often trivialise menstrual disorders (Verhoef et al. 2021), or they are not addressed in the sporting environment by coaches or medical staff (Laske et al. 2024; McHaffie et al. 2022; Verhoef et al. 2021). Discussing these issues and providing adequate support and medical care for female athletes who experience menstrual irregularities can be crucial for optimising their health and performance (Verhoef et al. 2021). The main barriers to communicating about the MC are a lack of knowledge, discomfort in discussing what is often considered a taboo topic, and a lack of evidence‐based and sport‐specific menstrual health education materials and strategies (Höök et al. 2021; McGawley et al. 2023). The coach's gender may intensify these barriers (Solli et al. 2020). Male coaches may avoid discussing menstrual health due to a lack of personal experience and to not infringe upon female athletes' privacy (Höök et al. 2021). Male coaches also report feeling unsure about how their female athletes wish to communicate about the MC and associated symptoms, and best practice guidelines on how to communicate about the MC in the coach–athlete relationship are lacking (Clarke et al. 2021). Female athletes often feel unsure, awkward and/or embarrassed about addressing MC‐related issues because they cannot assess how their coach will react or utilise this information (Brown et al. 2021).

In addition to uncertainty about how communication between the coach and the female athlete should take place, knowledge about the MC is often deemed insufficient (Solli et al. 2020). For example, Larsen et al. (2020) surveyed 189 Australian female athletes using a 13‐item questionnaire covering MC and oral contraceptive (OC) topics and reported a mean knowledge score of 36%, with only 33% ± 23 correct responses to questions about the MC. Johnson (2008) conducted a study involving 207 coaches from the south‐eastern United States of America to assess their knowledge, specific facets, and views on the MC and OC. The questionnaire development was based on focus group interviews with coaches and athletes, following an evaluation by gynaecology experts. The results showed that female coaches scored significantly higher than male coaches on the MC and OC knowledge test (61% vs. 50%). Mkumbuzi et al. (2023) studied familiarity with the MC and OCs within African women's football, employing a questionnaire adapted from various sources, including Larsen et al. (2020), Brown et al. (2021), and the Menstrual Attitudes Questionnaire (Brooks‐Gunn and Rouble 1986). The results indicated a lack of MC knowledge among female athletes, coaches, healthcare providers, and referees.

An MC questionnaire may be the optimal means of identifying specific knowledge deficits in this field, offering a simple, cost‐effective and efficient approach (Bauhaus et al. 2023). Currently, most MC knowledge surveys in the sports context employ a limited number of specific questions (Johnson 2008; Höök et al. 2021; Larsen et al. 2020; Mkumbuzi et al. 2023). For example, they may enquire about the definition of amenorrhoea, the names of female ovarian hormones that fluctuate throughout the MC, the average duration of a menstrual bleeding period, the average duration of one complete MC and the age at which the majority of females first menstruate (Larsen et al. 2020; Johnson 2008). Additional items seem necessary to map the physiology of the MC in detail. Moreover, selecting suitable items via the item difficulty index and item–total correlation procedures remains unexplored. Therefore, this study aimed to develop and validate a menstrual cycle knowledge questionnaire (MCKQ), encompassing knowledge about MC physiology, menstrual irregularities and MC‐related symptoms. This type of questionnaire would facilitate the assessment of MC education initiatives, thereby enabling the development of more effective and targeted educational strategies based on identified knowledge gaps.

## 2 Methods

**FIGURE 1:**
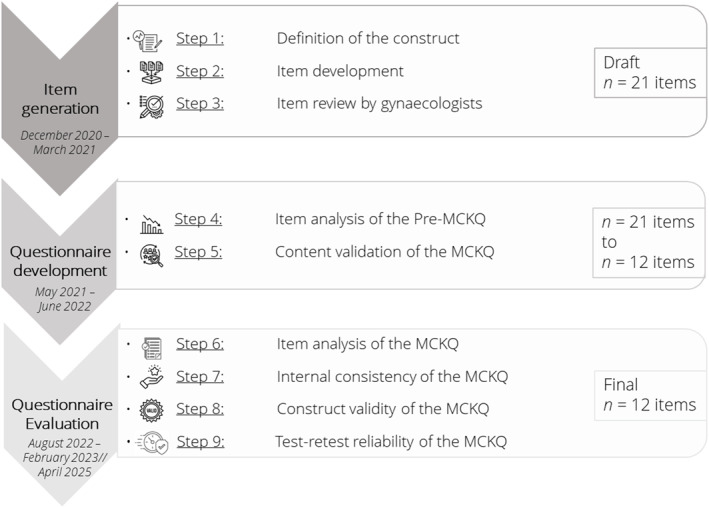
An overview of the three phases (9 steps) of developing and validating the 12‐item menstrual cycle knowledge questionnaire (MCKQ).

The development and validation of the MCKQ in the German language were mainly based on selected studies—Boateng et al. 2018; Heikkilä et al. 2018; Parmenter and Wardle 2000—led to nine steps, which were organised into three phases (Figure 1): item generation (Steps 1–3), questionnaire development (Steps 4 and 5) and questionnaire evaluation (Steps 6–9). The methods adopted within the nine steps are presented in the following subsections.

Before piloting the Pre‐MCKQ and testing the final 12‐item MCKQ with female athletes and coaches, the Ethics Committee of the Institute for Applied Training Science (ER_2021.22.06_7, ER_2022.01.17_12) approved the study. At each step, participants provided their informed consent after receiving all necessary information (e.g., questionnaire topics, procedures, data protection and confidentiality). Written parental consent was obtained for female athletes under the age of 16. Data were analysed using IBM SPSS for Windows, version 23 (IBM Corp., Armonk, NY, USA) and MS Excel (Mircosoft Corp, Redmond, WA, USA).

Step 1 *Definition of the construct.*

The first step entailed conducting a literature review to identify the areas to be covered when developing knowledge questions about the MC. The included domains were (1) basic knowledge about physiological MC processes, (2) menstrual irregularities and (3) MC‐related symptoms.

Step 2 *Item development.*

The aim of this step was to identify general knowledge items about the MC in the three identified areas of interest and to formulate the items to be able to differentiate between more and less informed athletes and coaches. Between December 2020 and March 2021, existing questionnaires and items from relevant publications in English and German were evaluated to discern whether the content was suitable for inclusion in an initial development questionnaire (Pre‐MCKQ). In addition, three experienced gynaecologists (2 female and 1 male aged 30–44 years) formulated items relating to the three areas of interest. The Pre‐MCKQ consisted of 21 items, and the detailed item development information, response formats and item origin are presented in Supporting Information S1 (Table S1). The questionnaire was designed in a deductive manner, and items were generated using a fixed response format and a single‐choice response option. Following Parmenter and Wardle's (2000) recommendation, a ‘don't know’ option was included in the response format to reduce the risk of participants guessing the correct answer when uncertain.

Step 3 *Item review by gynaecologists.*

The gynaecologists who participated in Step 2 analysed the Pre‐MCKQ in an online group discussion and assessed each item's content accuracy and language clarity. Based on their feedback, 12 items were reworded and nine items did not need to be improved.

Step 4 *Item analysis of the Pre‐MCKQ.*

The sample size calculation for piloting the Pre‐MCKQ was based on the ‘rule of thumb’ (Rosi et al. 2020), which states that the ratio between the number of participants (*n*) and the number of questionnaire items (*p*) should be at least ten. Because this questionnaire was comprised 21 items, at least 210 participants were targeted, including people similar to those for whom the measure is intended.

Recruitment for this step was conducted between May and July 2021 through various channels, including WhatsApp, sports clubs, emails, social media platforms (particularly sports groups on Facebook) and posters at the Sports Science Campus at Leipzig University. Female athletes who regularly participated in sports, and coaches, who worked voluntarily for a sports club, were targeted (elite athletes were excluded, because they were targeted for recruitment in Step 6), and all participants were required to be over the age of 16 years. A total of 325 recreational female athletes (age: *M* = 25.5 years and SD = 7.1) and 46 coaches (27 female, 59%; 19 male, 41%; and age: *M* = 28.2 years and SD = 6.9) participated in the Pre‐MCKQ, which was administered using the online survey platform SoSci Survey (Leiner 2025). Demographic questions covered participants' sex, age, first menstrual bleeding (for female participants), education, marital status, employment status, and sports‐related information, including sports discipline, weekly training volume, and coaching qualification level (e.g., C/B/A licence, diploma coach, or sports science studies). The Pre‐MCKQ items were subsequently analysed to determine what to include in the final version of the questionnaire (the MCKQ). Single items were analysed using the item difficulty index (i.e., percentages of correct answers for each question) and the item–total correlation (i.e. point–biserial correlations between the score for each question and the total score). According to Kline (2013), the item difficulty index of a questionnaire should be between 20% (below this indicates that the questions are too difficult) and 80% (above this indicates that the questions are too easy). The minimum item‒total correlation is usually set to *r* = 0.20 (Kline 2013), with higher values corresponding to stronger correlations. Items were divided into the following item–total correlation categories (Musa et al. 2018): poor (*r* < 0.20), acceptable (*r* = 0.20–0.29), good (*r* = 0.30–0.39) and excellent (*r* ≥ 0.40). Based on this analysis, nine items were excluded from the Pre‐MCKQ. This resulted in the 12‐item MCKQ.

Step 5 *Content validation of the MCKQ.*

Eighteen experts (17 female and 1 male, age: *M* = 38.8 years and SD = 11.4) including seven gynaecologists, five biologists, four sports physicians and two sports scientists with a particular interest in gynaecological health were invited through the authors' networks to form an expert panel to review the MCKQ. Each expert was asked to rate the 12‐item MCKQ using a 6‐point Likert scale on the following two dimensions: clarity (‘not at all clear’ = 1 to ‘very clear’ = 6) and relevance (‘not at all relevant’ = 1 to ‘very relevant’ = 6) (Ollesch et al. 2018). They were also allowed to comment freely on each MCKQ item. The validation of the MCKQ was conducted using an online questionnaire on SoSci Survey (Leiner 2025) between May and June 2022. The order of presentation of the MCKQ items was randomised.

The content validity index (CVI) was assessed using two different approaches based on the experts’ assessments of the relevance of each item. Firstly, their agreement on each item in the MCKQ was assessed using the item‐level CVI (I‐CVI), which is calculated by dividing the number of experts who rate an item as relevant (in this case, with a score of 4–6 on a 6‐point Likert scale, Ollesch et al. 2018) by the total number of experts. I‐CVI of 1.0 indicates full agreement among the experts. The scale‐level CVI (S‐CVI) reflects the experts' overall agreement with the entire questionnaire and is calculated by averaging the I‐CVI scores. A questionnaire's content validity is typically acceptable if the S‐CVI is 0.80 or higher (Bolarinwa 2015; Polit et al. 2007).

Those responding to the questionnaire were invited to participate in a videoconference, which lasted approximately 1.5 h, while the remaining questions were discussed. Five experts accepted. One of the authors (KF) moderated the discussion, presented all the comments on the items and ensured that participants were allowed to express themselves freely. Each item was presented to the panel during the discussion, and the moderator facilitated the discussion. Panel members had the opportunity to suggest changes or add clarifications to better reflect the group consensus. The moderator summarized panel feedback to refine statements as needed. This process resulted in the final version of the 12‐item MCKQ.

Step 6 *Item analysis of the MCKQ.*

Between August 2022 and February 2023, 562 German elite female athletes and 170 coaches (55 female, 32%; 114 male, 67% and 1 other) were recruited through national sports federations, Athleten Deutschland e.V. (Germany's independent Athletes' Association), Trainerakademie Köln des Deutschen Olympischen Sportbundes e.V. (Cologne Coaches’ Academy of the German Olympic Sports Federation), Berufsverband für Trainer/innen im deutschen Sport (Professional Association of Coaches in Germany) and via social media. Female athletes could participate in the survey if they were (i) at least 14 years old, (ii) had squad status in a German Olympic Sports Federation (DOSB) or were a member of a German regional squad, (iii) completed at least 5 h sport per week, (iv) regularly participated in competitions and (v) had a near‐native level of German. Coaches could participate in the survey if they were (i) at least 18 years old, (ii) currently coached or had coached female athletes with a national or regional squad, (iii) had at least one year coaching experience and (iv) had a near‐native level of German. Data were collected via an anonymous online survey with SoSci Survey (Leiner 2025). In addition to the final 12‐item MCKQ responses, demographic information was collected, including sex, age, age at first menstrual bleeding (for the female athletes), education, marital status, employment status, and sport‐related information including sports discipline, experience in primary sports, squad status, competition level, weekly training volume, number of weekly training sessions, sex of the coaching staff, sex distribution in the coaching team/training group, and level of coach qualification (e.g., C/B/A licence, diploma coach or study of sports science).

Answers from the MCKQ were used to reassess the item difficulty index and item–total correlation.

Step 7 *Internal consistency of the MCKQ.*

Cronbach’s alpha was calculated to assess the internal consistency of the MCKQ. Because intercorrelations between test items are maximised when all items measure the same construct, Cronbach’s alpha is considered an indirect measure of the extent to which a group of items measures a particular unidimensional latent construct (Hajjar 2018). According to the principles of classical test theory, a minimum threshold of 0.70 is generally recommended for satisfactory internal consistency (Parmenter and Wardle 2000).

Step 8 *Construct validity of the MCKQ.*

Construct validity was assessed by comparing participants' knowledge scores, calculated by assigning 1 point for correct answers and 0 points for incorrect or ‘don't know’ answers on the MCKQ. Previous research suggests that female coaches may have higher knowledge scores (Johnson 2008). In addition, it was expected that participants who rated their MC knowledge higher (rated on a 6‐point Likert scale from 1 = ‘excellent’ to 6 = ‘insufficient’) would achieve higher knowledge scores (Engelberg 2015). A one‐way ANOVA followed by a post hoc Bonferroni correction was used to analyse the differences among the knowledge scores of the female athletes, the female coaches and the male coaches and the self‐rated MC knowledge.

Step 9 *Test–retest reliability.*

To assess the test–retest reliability of the MCKQ, 64 sport science students at Leipzig University (34 female [53%], 29 male [45%] and 1 identified as ‘other’ [2%]; *M* = 21.3 years and SD = 1.7) completed the questionnaire twice (at Time 1 and Time 2) in April 2025, with a one‐week interval between assessments. Reliability was evaluated using Spearman's correlation coefficients and intraclass correlation coefficients (ICC), based on a two‐way mixed‐effects model with absolute agreement (Pai et al. 2022). Spearman correlation coefficients can be interpreted as follows: very weak (0.00–0.19), weak (0.20–0.39), moderate (0.40–0.69), strong (0.70–0.89) and very strong (0.90–1.00; De Ridder et al. 2021). In addition, Bland–Altman plots with limits of agreement were generated to assess the agreement between the two measurements. A paired‐samples *t*‐test was conducted to identify any potential systematic bias (Pai et al. 2022), and statistical significance was set at *p* < 0.05.

## 3 Results

### 3.1 Item Analysis of the Pre‐MCKQ

Table 1 shows all 21 items with item difficulty and item–total correlation scores for the Pre‐MCKQ. The item difficulty index was lower than 20% for two items (MC_18 and MC_19), while five items (MC_01, MC_03, MC_05, MC_06 and MC_13) had a difficulty index of more than 80%, and none of the items were correctly answered by all participants. These items were removed, except item MC_01 (item difficulty 89%), which remained as an ‘opener’ to provide an easy start to the questionnaire. Also, item MC_06 (item difficulty 89%) remained because it addressed basic knowledge about an essential mechanism of menstrual bleeding. Four items (MC_02, MC_08, MC_09 and MC_20) with poor discriminating ability (*r* < 0.20) were also excluded. A total of nine items with poor item–total correlation (*n* = 4; *r* < 0.20) or an inappropriate difficulty index (*n* = 5; < 20%/> 80%) were excluded from the questionnaire, which resulted in a 12‐item MCKQ.

**TABLE 1:** Item descriptions, difficulty and item–total correlation scores and reasons for item exclusion for the initial 21‐item development Menstrual Cycle Knowledge Questionnaire (Pre‐MCKQ).

Item no.	Item	Item difficulty index [%]	SD	Item–total correlation	Reason for exclusion
MC_01*	At what age, on average, do women get their period (menarche) for the first time?	**0.89**	**0.31**	**0.06**	
MC_02	At what age, on average, do women have their last period (menopause)?	0.61	0.49	0.05	Item–total correlation
MC_03	The average length of a menstrual cycle is …	0.92	0.27	0.32	Item difficulty
MC_04*	**When is the first day of the menstrual cycle?**	**0.62**	**0.49**	**0.40**	
MC_05	What information about the length of the bleeding period is correct?	0.86	0.35	0.25	Item difficulty
MC_06*	**What is the cause of bleeding?**	**0.89**	**0.31**	**0.37**	
MC_07*	**On which day does ovulation occur?**	**0.79**	**0.41**	**0.37**	
MC_08	Which of the following is NOT likely to cause your period to be late?	0.38	0.49	0.22	Item–total correlation
MC_09	What is the average amount of blood loss during a period?	0.42	0.49	0.16	Item–total correlation
MC_10*	**Why does blood come?**	**0.75**	**0.44**	**0.31**	
MC_11*	What are the primary reasons for menstrual pain during bleeding?	**0.74**	**0.44**	**0.33**	
MC_12*	**What is amenorrhoea?**	**0.36**	**0.48**	**0.46**	
MC_13	What does the abbreviation PMS stand for?	0.83	0.38	0.38	Item difficulty
MC_14*	**When does the body temperature increase during the menstrual cycle?**	**0.57**	**0.50**	**0.37**	
MC_15*	**Which of the symptoms may be associated with premenstrual syndrome (PMS)?**	**0.70**	**0.46**	**0.26**	
MC_16*	**What is the correct order of phases during the menstrual cycle?**	**0.54**	**0.50**	**0.47**	
MC_17*	**Where is the menstrual cycle controlled?**	**0.44**	**0.50**	**0.45**	
MC_18	Which hormone peak triggers ovulation?	0.13	0.33	0.29	Item difficulty
MC_19	Which hormone is responsible for producing PMS symptoms?	0.15	0.36	0.28	Item difficulty
MC_20	Which of the following are the effects of increased oestrogen levels in the follicular phase of the menstrual cycle?	0.26	0.44	0.15	Item–total correlation
MC_21*	**Which statement is correct?**	**0.28**	**0.45**	**0.28**	

*Note:* After the pilot phase, the final version of the questionnaire kept bold items labelled with an asterisk.

Abbreviations: PMS = premenstrual syndrome; SD = standard deviation.

### 3.2 Content Validation of the MCKQ

Item MC_21 did not display as intended in the online questionnaire on SoSci Survey due to an incorrect randomization setting. Therefore, only 11 items could be rated by the 18 experts. All items were rated as highly relevant (*M* = 5.4, SD = 0.8) and highly clear (*M* = 5.4, SD = 1.0), see Supporting Information S1: Table S2 for details. The results of the I‐CVI and the S‐CVI for each item are shown in Table 2. The experts rated the questionnaire with an S‐CVI of 97%. Further linguistic revisions (e.g., rephrasing items/answers) of items are presented in Supporting Information S1: Table S3. Minor adjustments were made to improve the clarity and comprehensibility of the questions. The comments and suggestions for improvement were implemented by revising the wording of items MC_01, MC_04, MC_06, MC_10, MC_11, MC_12; MC_14, MC_15, MC_16 and MC_17.

**TABLE 2:** Item content validity index (I‐CVI) and scale content validity index (S‐CVI) scores for 11 of the 12 Menstrual Cycle Knowledge Questionnaire (MCKQ) items as rated by *n* = 18 experts on a scale of 1 (not relevant at all) to 6 (very relevant).

	Expert	Expert	Expert	Expert	Expert	Expert	Expert	Expert	Expert	Expert	Expert	Expert	Expert	Expert	Expert	Expert	Expert	Expert	*n* = experts in agreement	I‐CVI
	1	2	3	4	5	6	7	8	9	10	11	12	13	14	15	16	17	18		
MC_01	6	6	5	5	5	6	5	6	4	5	6	6	6	6	6	6	6	3	17	0.94
MC_04	6	5	5	4	6	6	6	6	4	6	5	6	6	6	6	6	4	3	17	0.94
MC_06	6	6	4	5	5	6	6	6	3	4	5	6	6	6	6	6	6	4	17	0.94
MC_07	6	6	5	5	6	6	6	6	4	6	6	6	6	5	6	4	6	5	18	1.00
MC_10	6	5	5	6	6	6	6	6	5	5	5	6	6	4	6	6	6	4	18	1.00
MC_11	6	6	6	5	5	6	4	4	5	5	4	5	6	6	6	4	6	4	18	1.00
MC_12	6	5	6	5	6	6	6	6	5	6	6	6	6	5	6	6	5	5	18	1.00
MC_14	5	6	6	4	6	6	6	6	4	6	6	5	6	4	6	4	5	3	17	0.94
MC_15	6	4	5	5	5	6	6	6	5	5	6	5	5	6	6	4	4	3	17	0.94
MC_16	6	6	6	5	6	6	6	6	5	6	5	6	6	4	6	5	5	4	18	1.00
MC_17	6	6	4	5	5	6	3	6	5	5	5	6	6	5	6	6	5	5	17	0.94
																			S‐CVI	0.97

### 3.3 Item Analysis of the MCKQ

Descriptive (including demographic and educational) information for the female athletes and coaches who completed the final 12‐item MCKQ is presented in Table 3. Table 4 shows the final MCKQ with response options (presented in English [translated] and German [original]), and item difficulty and item–total correlation scores. The mean difficulty index was 57% (SD: 18; range: 30%–93%). Item MC_01 was the easiest for the participants to answer, and item MC_12 was the most difficult. The mean item–total correlation was *r* = 0.35 (SD: 0.13; range: from *r* = −0.01 to *r* = 0.52).

**TABLE 3:** Descriptive (including demographic and educational) information for the female athletes and female and male coaches who completed the Menstrual Cycle Knowledge Questionnaire (MCKQ).

Characteristics	Female athletes (*n* = 562)	Female coaches (*n* = 55)	Male coaches (*n* = 114)
M ± SD (min—max)			
Age (years)	21.0 ± 5.1 (14–43)	34.0 ± 10.7 (18–65)	39.3 ± 10.6 (19–70)
Menarche age (years, *n* = 544)	13.4 ± 1.6 (9–19)		
Weekly training (hours)	17.6 ± 7.3 (5–45)		
Years in primary sport (athletes) or coaching experience (coaches)	11.1 ± 5.1 (0–35)	10.7 ± 9.6 (1–40)	13.8 ± 9.0 (1–38)
Average age of the female athlete training group (years)		15.1 ± 5.0	19.9 ± 4.7
*n* (%)			
Highest educational level			
No school‐leaving qualification	74 (13)		
9th grade	13 (2)		2 (2)
10th grade	101 (18)	3 (6)	6 (5)
12th/13th grade	259 (46)	10 (18)	19 (17)
Apprenticeship	16 (3)	2 (4)	5 (4)
Polytechnic degree	12 (2)	2 (4)	4 (4)
Bachelor, master	85 (15)	37 (67)	75 (66)
Doctorate (PhD)	2 (0)	1 (2)	3 (3)
Competition level			
International	408 (73)		
National	130 (23)		
Regional	7 (1)		
Other	17 (3)		
Sport discipline			
Endurance	180 (32)	30 (55)	51 (45)
Power	78 (14)	8 (15)	29 (25)
Aesthetic	45 (8)	4 (7)	
Combat	85 (15)	4 (7)	10 (9)
Ball games	123 (22)	8 (15)	22 (19)
Other	51 (9)	1 (2)	2 (2)

*Note:* The coach with sex ‘other’ (*n* = 1) is not listed in the table to ensure anonymity.

Abbreviations: *M* = mean; max = maximum; min = minimum; SD = standard deviation.

**TABLE 4:** Item descriptions (with English translations and the original German text) and difficulty and item–total correlation scores for the final 12‐item Menstrual Cycle Knowledge Questionnaire (MCKQ).

Item no.	Item (English)	Item (German)	Item difficulty index [%]	SD [%]	Item–total correlation
MC_1	At what age, on average, do girls get their period (menarche) for the first time? 9–11 years of age **11–14 years of age** 15–17 years of age > 17 years of	In welchem alter haben junge frauen im durchschnitt ihre erste regelblutung (menarche)? 9. 11. Lebensjahr **11. 14. Lebensjahr** 15. 17.Lebensjahr > 17. Lebensjahr	0.93	0.26	−0.01
MC_4	When is the first day of the menstrual cycle? The day ovulation occurs The last day of the period The first day after the period **The first day of the period**	Wann beginnt ein menstruationszyklus? Tag des eisprungs Letzter Tag der regelblutung Erster Tag nach der regelblutung **Erster Tag der regelblutung**	0.51	0.50	0.35
MC_6	What is the cause of bleeding? **Rejection of the ‘old’ endometrium** Termination of pregnancy Functionality check of the uterus Elimination of defective blood cells	Was ist bzw. Wozu dient die regelblutung? **Abstoßung der ‘alten’ gebärmutterschleimhaut** Schwangerschaftsabbruch Funktionstüchtigkeit der gebärmutter Ausscheidung kaputter blutzellen	0.86	0.35	0.33
MC_7	If the egg has not been fertilised.... Then, the period starts about one week after ovulation **Then, the period starts about two weeks after ovulation** Then, the period starts about three weeks after ovulation Then, the period starts about four weeks after ovulation	Wenn die eizelle nicht befruchtet wurde… Dann setzt ca. Eine woche nach dem eisprung die regelblutung ein **Dann setzt ca. Zwei wochen nach dem eisprung die regelblutung ein** Dann setzt ca. Drei wochen nach dem eisprung die regelblutung ein Dann setzt ca. Vier wochen nach dem eisprung die regelblutung ein	0.56	0.50	0.36
MC_10	Which mechanisms are reasonable for menstrual bleeding? Blood supply from the uterus (womb) is diverted Micro‐tears during ovulation **Drop in hormones leads to shedding of uterine lining** Hormone constellation increases blood production	Durch welche mechanismen kommt es zur regelblutung? Blutversorgung der gebärmutter (uterus) wird abgeleitet Mikroeinrisse beim eisprung (ovulation) **Abfall der hormone führt zu abstoßen der gebärmutterschleimhaut** Hormonkonstellation führt zu erhöhter blutproduktion	0.65	0.48	0.45
MC_11	What are the primary reasons for menstrual pain during bleeding? **Uterine contractions** Ovulation Opening of the cervix Inflammatory reaction	Was sind primäre gründe für schmerzen während der regelblutung? **Gebärmutterkontraktionen** Eisprung (ovulation) Öffnung des muttermundes Entzündungsreaktion	0.67	0.47	0.40
MC_12	What is amenorrhoea? **Absence of a period** Intermenstrual bleeding Development of the ovaries Painful proliferation of the tissue of the endometrium	Was versteht man unter amenorrhö? **Ausbleiben der regelblutung** Zwischenblutungen Aufbau der eierstöcke Schmerzhafte wucherung vom gewebe der gebärmutterschleimhaut	0.30	0.46	0.41
MC_14	What triggers the body's temperature increase during the menstrual cycle? **Ovulatio**n The beginning of the period The end of the period The basal body temperature does not change	Was löst den anstieg der körperkerntemperatur im menstruationszyklus aus? **der eisprung (ovulation)** der beginn der regelblutung Das ende der regelblutung die temperatur verändert sich nicht	0.50	0.50	0.48
MC_15	Which are not accompanying symptoms of premenstrual syndrome (PMS)? Irritability. Emotional upset. Headache Chest pain. Fatigue. Increased appetite Constipation. Flatulence. Water retention **Urine leakage. Fever episodes. Colour change of nipples**	Was sind keine begleitsymptome des prämenstruellen syndroms (PMS)? Reizbarkeit, emotionale verstimmungen, kopfschmerzen Brustschmerzen, müdigkeit, gesteigerter appetit Verstopfungen, blähungen, wassereinlagerungen **Urinverlust, fieberschübe, farbliche veränderung der brustwarzen**	0.58	0.49	0.36
MC_16	What is the correct order of phases during the menstrual cycle? Bleeding. Luteal phase. Ovulation. Follicle phase **Bleeding. Follicle phase. Ovulation. Luteal phase** Luteal phase. Bleeding. Ovulation. Follicle phase Ovulation. Follicular phase. Bleeding. Luteal phase	Was ist die korrekte zeitliche abfolge der phasen des menstruationszyklus? Regelblutung, gelbkörperphase (lutealphase), eisprung (ovulation), follikelreifung **Regelblutung, follikelreifung, eisprung (ovulation), gelbkörperphase (lutealphase)** Gelbkörperphase (lutealphase), regelblutung, eisprung (ovulation), follikelreifung Eisprung (ovulation), follikelreifung, regelblutung, gelbkörperphase (lutealphase)	0.38	0.49	0.52
MC_17	Where is the menstrual cycle controlled? Pituitary gland (hypophysis). Ovaries (ovaries). Vagina (vagina) Hypothalamus. Ovaries (ovaries). Pancreas (pancreas) Hypothalamus. Pituitary gland (hypophysis). Vagina (vagina) **Hypothalamus. Pituitary gland (hypophysis). Ovaries (ovaries)**	Wodurch wird der menstruationszyklus gesteuert? Hirnanhangsdrüse (hypophyse), eierstöcke (ovarien), scheide (vagina) Hypothalamus, eierstöcke (ovarien), bauchspeicheldrüse (pankreas) Hypothalamus, hirnanhangsdrüse (hypophyse), scheide (vagina) **Hypothalamus, hirnanhangsdrüse (hypophyse), eierstöcke (ovarien)**	0.37	0.48	0.41
MC_21	Which statement about hormones is false? Oestrogen has a muscle‐building (anabolic) effect. Progesterone has a muscle‐degrading (catabolic) effect. **Testosterone is exclusively produced in the male body.** The increase in luteinising hormone (LH peak) triggers ovulation.	Welche aussage über hormone ist falsch? Östrogen wirkt muskelaufbauend (anabol). Progesteron wirkt muskelabbauend (katabol). **Testosteron wird ausschließlich im männlichen körper gebildet.** Anstieg des luteinisierenden hormons (LH‐peak) löst den eisprung aus.	0.50	0.50	0.20

*Note:* The correct answer is in bold font. The answer format ‘don't know’ was added to each item.

Abbreviations: LH = luteinising hormone; PMS = premenstrual syndrome; SD = standard deviation.

### 3.4 Internal Consistency and Construct Validity of the MCKQ

Cronbach's alpha for the MCKQ was 0.73, indicating internal consistency of the questionnaire. The knowledge test scores significantly differed between groups (*F* (2,728) = 8.59, *p* < 0.01, *ηp* ^*2*^ = 0.02 and *n* = 731), with female coaches (*M* = 8.22 and SD = 2.64) scoring significantly higher than male coaches (*M* = 6.40 and SD = 2.82; *p* < 0.01) and female athletes (*M* = 6.75 and SD = 2.74; *p* < 0.01). No significant differences were found between male coaches and female athletes (*p* = 0.65). In addition, knowledge test scores differed significantly between participants who rated their MC knowledge as excellent and those who rated it as insufficient (*F* (5,726) = 38.45, *p* < 0.001, *ηp* ^*2*^ = 0.21 and *n* = 732). Table 5 provides detailed information on group differences.

**TABLE 5:** Construct validity across different groups (female athletes, female and male coaches) and knowledge levels (1 = ‘excellent’ to 6 = ‘inadequate’) for the final 12‐item Menstrual Cycle Knowledge Questionnaire (MCKQ).

Group	*n* (%)	Knowledge score M ± SD
Female athletes	562 (77)	6.75 ± 2.74^a^
Female coaches	55 (7)	8.22 ± 2.64
Male coaches	114 (16)	6.40 ± 2.82^a^
Knowledge rating		
1 Excellent	30 (4)	10.23 ± 1.76^b^
2 Good	227 (31)	7.96 ± 2.48^c^
3 Satisfactory	300 (41)	6.57 ± 2.53^d^
4 Pass	136 (19)	5.40 ± 2.56
5 Poor	35 (5)	4.43 ± 2.16
6 Insufficient	4 (0)	3.50 ± 3.00

Abbreviations: M = mean; SD = standard deviation.

^a^ Significantly different from female coaches (*p* < 0.01).

^b^ Knowledge—Rating ‘1’ (very good) significantly different to all other scores (*p* < 0.01).

^c^ Knowledge—Rating ‘2’ significantly different to ‘3’, ‘4’ and ‘5’ (*p* < 0.01) and ‘6’ (*p* < 0.05).

^d^ Knowledge—Rating ‘3’ significantly different to ‘4’ and ‘5’ (*p* < 0.01).

### 3.5 Test–Retest Reliability

Between Time 1 and Time 2, 70% of the 64 sport science students reported that they had not received any additional information related to the topic during the intervening week. The Spearman's rank correlation coefficient between Time 1 (*M* = 6.14, SD = 2.56) and Time 2 (*M* = 6.48, SD = 2.66) scores was *ρ* = 0.82 (*p* < 0.01), indicating a strong association. Similarly, the ICC (2,1) was 0.82 (95% CI: 0.72–0.89), reflecting strong agreement and suggesting that the MCKQ produces consistent scores over time. A paired‐samples *t*‐test indicated that the Time 1 and Time 2 scores were not significantly different (*p* = 0.08). A Bland–Altman analysis revealed a mean difference between Time 1 and Time 2 scores of 0.34 points, with 95% limits of agreement ranging from −0.04 to 0.72.

## 4 Discussion

This study aimed to develop and validate a German language knowledge questionnaire that assesses female athletes' and coaches' knowledge of the MC. In particular, the questionnaire aimed to measure knowledge about basic physiological MC processes, menstrual irregularities and MC‐related symptoms relevant to training.

The MCKQ contributes to the existing literature by providing an updated and comprehensive tool for assessing knowledge of the MC. Our MCKQ instrument is short, which is particularly beneficial in elite sports settings, where athletes and coaches have limited time (Behnke et al. 2019). Also, participants show higher concentration when measurement instruments are short (Bolarinwa 2015).

The MCKQ builds on the foundations of the only, to our knowledge, other validated questionnaire in this area (Johnson 2008). This previous questionnaire was developed for coaches mainly through focus group interviews with coaches and athletes. Unlike Johnson (2008), our instrument includes specific items about the phases of the MC and hormonal changes during the MC. Consistent with Johnson's (2008) questionnaire, the MCKQ included a ‘don't know’ response option for items. Participants with lower knowledge scores were more likely to select the ‘don't know’ option, confirming a significant negative correlation between knowledge score and frequency of ‘don't know’ responses (*r* = −0.78, *p* < 0.01). This suggests that including ‘don't know’ may have decreased the likelihood of participants guessing (Parmenter and Wardle 2000).

Our questionnaire demonstrated high content validity, as assessed by the expert panel consisting of 18 relevant experts, that is, gynaecologists, biologists, sports physicians and sports scientists. Female coaches scored significantly higher on knowledge about the MC than male coaches and female athletes, which indicates validity and is consistent with previous research (Johnson 2008) showing female coaches to be better informed about the MC than male coaches. The lack of personal experience and education regarding the relevance of the MC for training (Höök et al. 2021) may explain male coaches' lower MC knowledge scores. One possible reason for the lower knowledge scores among female athletes could be the lack of structures to adequately inform them about the MC during the training process (Höök et al. 2021). Additionally, many female athletes have reported that the MC does not influence their training, which may reflect a lack of awareness or limited knowledge about the topic (Solli et al. 2020). Also indicating content validity, participants with a higher self‐assessment of their knowledge of the MC had significantly higher scores than those with a lower self‐assessment of their knowledge of the MC (Engelberg 2015). Based on our results, the 12‐item MCKQ appears to be a reliable and valid instrument for measuring theoretical MC knowledge. Further research and testing of the MCKQ in different populations and contexts would provide valuable insights into its robustness and applicability. In future, the MCKQ could also be used to determine whether athletes with a higher level of knowledge already consider the MC when planning, executing and documenting their training and/or discussing MC‐related symptoms and problems with their coach(es).

Assessing test–retest reliability for a knowledge‐based questionnaire presents specific challenges, particularly due to the potential for learning effects between time points (Pai et al. 2022). To minimize this risk, we chose a short retest interval of one week (De Ridder et al. 2021). Notably, 70% of participants reported not having received any additional information on the topic during that period, whereas the remaining 30% stated that they received only minimal or incidental information. This supports the assumption of relatively stable knowledge conditions. The statistical analyses used to evaluate agreement, including a Spearman's rank correlation coefficient, ICC, paired *t*‐test and Bland–Altman analysis, consistently indicated strong reliability of the questionnaire across time. To our knowledge, no other MC knowledge questionnaire has reported test–retest reliability data. Further MC knowledge test development should include test–retest reliability measurements to demonstrate the stability of a measure over time (Rattray and Jones 2007).

### 4.1 Limitations and Future Applications

At present, the MCKQ has only been validated in the German language. Translating the MCKQ to other languages would create possibilities for cross‐cultural comparisons. Future studies could focus on assessing the validity and reliability of the MCKQ in other languages.

Another limitation of this study is that none of the experts were able to rate item MC_21 in step 5 due to a programming error. However, the item was discussed in a subsequent online meeting with five members of the 18‐member expert group, and it was found to be clear and relevant, so no further changes were made. Nevertheless, item MC_21 showed poor item–total correlation (*r* = 0.20), indicating that response options to the item may not have been sufficiently clear. In future studies, item MC_21 should be further evaluated and a revised version could be tested.

We treated coaches and female athletes as one group while conducting the item analysis in steps 4 and 6, and while calculating the difficulty and item–total correlation scores, without considering the differences between the groups, which can be another limiting factor. In each case, female athletes comprised the largest proportion of participants (88% in step 4% and 77% in step 6). Therefore, the items selected may have been more suited to female athletes over coaches, and the item selection may have differed if all three groups (i.e., female athletes, female coaches and male coaches) had been analysed separately.

This study used internal consistency as a type of reliability. Cronbach's alpha was 0.73, which is lower than Johnson's (2008) measure, which had a value of 0.93. However, Cronbach's alpha values generally increase with the number of test items (Parmenter and Wardle 2000), and our instrument had half the number of items compared to Johnson's instrument.

A further limitation to our study is that no factor analysis was conducted to test validity. This was due to the diversity of aspects represented by the items and the varying difficulty levels of the items included in the questionnaire. A factor analysis would have been an opportunity to assess whether the questionnaire could be divided into different sub‐scales. However, the analysis of individual factors was not carried out due to the brevity of the questionnaire (only 12 items) and because several aspects of the MC are represented by only a single item.

### 4.2 Practical Implications

In addition to the possibility of using the questionnaire to descriptively assess the level of knowledge about MC in a German‐speaking population, the MCKQ could also be used to determine if a variety of intervention approaches are needed to meet individual educational needs. Pre‐ to post‐MCKQ intervention testing could also be used to measure the effectiveness of, evaluate and potentially improve MC educational programmes and formats. This could contribute to the continuous improvement of MC‐related educational materials and resources. The questionnaire can also be an excellent basis for discussion. It can provide a starting point for dialogue between coaches and athletes. The MCKQ was initially developed in sports context, but its content is designed to apply to a wide range of applications also outside of sports.

## 5 Conclusion

In conclusion, the 12‐item MCKQ appears to be a valid and reliable measurement tool that can be used to assess female athletes' and coaches' MC knowledge. Its brevity makes it practically useful in the elite sports context. It promises to be a valuable tool for assessing knowledge and evaluating interventions to enhance MC knowledge in sports. A useful next step could be to assess the validity and reliability of the MCKQ when translated into other languages.

## Author Contributions

K.F.: methodology, investigation, statistical analysis, visualization, writing – original draft, funding acquisition. E.M.K.: methodology, investigation, writing – review and editing, funding acquisition. L.N.: methodology, writing – review and editing. F.L.: writing – review and editing. K.M.: supervision, methodology, writing – review and editing. A.M.E.: supervision, methodology, writing – review and editing.

## Ethics Statement

The Ethics Committee of the Institute for Applied Training Science (ER_2021.22.06_7, ER_2022.01.17_12) approved the study.

## Consent

At each step, participants provided their informed consent after receiving all necessary information (e.g., questionnaire topics, procedures, data protection and confidentiality). Written parental consent was obtained for participants under the age of 16 years.

## Conflicts of Interest

The authors declare no conflicts of interest.

## Supporting information

Supporting Information S1

## Data Availability

The data sets generated and analysed during the current study are available from the corresponding author upon reasonable request.
