# Supplementary material for: The Development and Validation of a Questionnaire That Assesses Female Athletes’ and Coaches’ Knowledge of the Menstrual Cycle
Source: Eur J Sport Sci. 2025 Aug 23;25(9):e70019. doi: 10.1002/ejsc.70019 (PMC12374752; doi:10.1002/ejsc.70019)
Supplement: Supplementary file 1 — Supporting Information S1 [file EJSC-25-e70019-s001.pdf]

Table S1 An overview of the items included in the initial development Menstrual Cycle Knowledge Questionnaire (Pre-MCKQ) for female athletes and coaches (green text has been translated from German)

Item no.	Gynaecologist 1	Gynaecologist 2	Gynaecologist 3	Research of scientific and non-scientific questionnaires	Initial Item
MC_01	When in life does the period physiologically begin?	At what age do most girls get their period?		At about what age do most girls begin to menstruate?<br>a) age 9 years<br>b) age12years<br>c) age 15 years<br>d) age18years<br>e) I am not sure (Johnson, 2008)<br>At what age do most girls get their first period?<br>a) at about the age of 10<br>b) between 11 and 15 years<br>c) after the age of 15 (Johnson & Johnson, n.d.)	At what age, on average, do women get their period (menarche) for the first time?<br>- **Age 9-11**<br>- Age 11-14<br>- Age 15-17<br>- > Age 17
MC_02				At what age do women stop getting their period? (Benshaul-Tolonen et al., 2020)<br>What is the average age of menopause?<br>a) 40-49<br>b) 50-59 (Harvey-Jenner & Millington, 2023)	At what age, on average, do women have their last period (menopause)?<br>- Under 50 years<br>- **50-54 years**<br>- 55-59 years<br>- 60-64 years
MC_03	How long does a normal cycle last?	How long is a menstrual cycle?	How long is the average woman's cycle?	What is the average length of a menstrual cycle?<br>a) 20+/-5 days<br>b) 24+/-6 days<br>c) 28+/-7 days<br>d) 35+/-10 days (Warzecha et al., 2019)<br>How long does an average menstrual cycle last?<br>a) 7 days<br>b) 16 days<br>c) 28 days<br>d) 40 days<br>e) I don't know (Erdbeerwoche, 2017)<br>How long does a menstrual cycle normally last?<br>a) 21 to 35 days<br>b) 28 days<br>c) 23 to 35 days<br>d) 25 to 30 days (Krahlisch, 2013)<br>How long is a healthy cycle?<br>a) 28 days<br>b) less than 23 days	The average length of a menstrual cycle is ...<br>– 14 days<br>- 23 days<br>- **28 days.**<br>- 35 days.

				c) 23 to 35 days<br>d) more than 35 days (Trackle GmbH, n.d.)<br>How long is a normal menstrual cycle?<br>a) 21 days<br>b) 28 days<br>c) 35 days<br>d) all of the above (Begum, 2023)	
MC_04	When does the cycle begin?	When is the 1st day of the cycle?	Which days of the cycle make up menstruation?	What day is the first day of the menstrual cycle?<br>a) The last day of menstrual bleeding.<br>b) The day when ovulation occurs.<br>c) The first day of menstrual bleeding.<br>d) It depends on the patient's choice. (Warzecha et al., 2019)<br>When does the female cycle start?<br>a) On the day of ovulation<br>b) On the last day of menstruation<br>c) On the first day of menstruation<br>d) On the day with the highest temperature measurement (Trackle GmbH, n.d.)	When is the first day of the menstrual cycle?<br>- **1st day of the period**<br>- Day of ovulation<br>- Last day of the period<br>- First day after the period
MC_05	How long do you normally bleed for?	How long does a period last?	How long is the average duration of the "menstrual period" (menses)?	What is the average duration of a menstrual bleeding period?<br>a) 2 days<br>b) 5days<br>c) 9 days<br>d) 13days<br>e) I am not sure (Johnson, 2008)<br>How long does the average menstrual bleeding last and what is the average blood loss?<br>a) About 3-5 days and 30-70 ml.<br>b) About 5-7 days and less than 30 ml.<br>c) About 7-10 days and less than 30ml<br>d) It doesn't matter how long the menstruation lasts. (Warzecha et al., 2019)<br>How long does menstruation normally last?<br>a) 2 to 7 days<br>b) At least 5 days<br>c) More than 10 days<br>d) 5 to 10 days (Krahlisch, 2013)	What information about the length of the bleeding period is correct?<br>- 3 days<br>- 5 days<br>- 7 days<br>- **All of the above (3-7 days)**
MC_06			What is menstrual bleeding and what is it for?		What is the cause of bleeding?<br>- **Rejection of the "old" mucosa**<br>- Termination of pregnancy<br>- Function check<br>- Cleansing the hormone balance

MC_07		When does ovulation occur?	Around which day of the cycle does ovulation take place?	On which days between two menses are you most prone to become pregnant?<br>a) At the beginning<br>b) At the middle<br>c) At the end<br>d) Whenever (Szücz et al., 2016)<br>On which day does ovulation occur?<br>a) Immediately after the end of menstruation.<br>b) Usually 14 days before the next period.<br>c) About the 20th day of the cycle (if it is regular, 25-30 days).<br>d) 7 days before the expected menstruation.<br>(Warzecha et al., 2019)	On which day does ovulation occur?<br>- On the first day of the cycle<br>- On the first day after your period<br>- **Approx. 14th day of cycle**<br>- Approx. 20th day of the cycle
MC_08	When does menstruation stop?			Which of the following is NOT likely to cause your period to be late?<br>a) Stress<br>b) Swimming<br>c) Weight loss<br>d) Illness (Your Fertility Journey, 2018)<br>What does NOT affect your period?<br>a) Stress<br>b) significant changes in weight<br>c) the pill<br>d) the weather (Mädchen.de, 2021)	Which of the following is NOT likely to cause your period to be late?<br>- Pregnancy<br>- Lack of energy<br>- High blood pressure<br>- **Jet lag**
MC_09	What is the normal blood loss?		How high do you estimate the average physiological blood loss per menstrual cycle?	What is the average amount of blood loss during a period?<br>a) a litre<br>b) 500 mls<br>c) 1-2 mls<br>d) 30-40 mls (Your Fertility Journey, 2018)<br>How much blood does the average woman lose during each period?<br>a) 2 Tbs<br>b) 1/3 cup<br>c) 1 cup (Begum, 2023)<br><br>On average, how much fluid does a woman release during her period?<br>a) About two tablespoons, or 40 millilitres<br>b) About five tablespoons, or 100 millilitres<br>c) About 250 millilitres<br>d) About half a litre (ABC Health & Wellbeing, 2017)	What is the average amount of blood loss during a period?<br>- 2 tablespoons<br>- **½ coffee cup**<br>- 1 coffee cup<br>- 1 bottle (0.5l)

MC_10	Why does blood come?				Why does blood come?<br>- **Drop in hormones leads to bleeding of the mucous membrane**<br>- Haemorrhages in the uterus are drained off<br>- Microcracks during ovulation<br>- Hormone constellation leads to increased blood production
MC_11	Why does menstrual pain occur?			What are period cramps?<br>a) When the muscular wall of the womb tightens<br>b) the natural shedding of the uterus wall (Harvey-Jenner & Millington, 2023)<br>What is the most common cause of period pain?<br>a) The release of an egg from the ovary<br>b) The contraction of muscles in the uterus<br>c) A build up of menstrual fluid in the uterus<br>d) A woman's imagination (ABC Health & Wellbeing, 2017)	What are the primary reasons for menstrual pain during bleeding?<br>- **Uterine contractions**<br>- Ovulation (ovulation)<br>- Opening of the cervix<br>- Psychosomatic causes
MC_12				What is amenorrhea?<br>a) a condition of the absence of menstruation<br>b) a condition of regular patterns of menstrual cycle functioning<br>c) a condition of irregular patterns of menstrual cycle functioning<br>d) a condition of painful menstruation<br>e) I am not sure (Johnson, 2008; Larsen et al., 2020)	What is amenorrhoea?<br>- **Absence of menstruation**<br>- Intermediate bleeding<br>- Structure of the ovaries<br>- Painful proliferation of the tissue of the uterine lining
MC_13		What is premenstrual syndrome?	What is premenstrual syndrome (PMS)?	What does the abbreviation PMS represent?<br>a) perimenstrual stage<br>b) postmenstrual shock<br>c) premenstrual syndrome<br>d) painful menstruation syndrome<br>e) I am not sure (Johnson, 2008)	What does the abbreviation PMS represent?<br>- **Physical and emotional discomfort a few days before your period**<br>- Mood high before menstruation<br>- Pain during menstruation<br>- Time of increased performance in the menstrual cycle

MC_14	At what time is the basal body temperature the highest?	When does the basal body temperature increase during the menstrual cycle?<br>a) During menstruation.<br>b) At the time of ovulation.<br>c) In the second phase of the menstrual cycle (after ovulation).<br>d) The cycle phase has no influence on basal body temperature. (Warzecha et al., 2019)<br>How does the 0.5 degree C higher temperature in the secretory phase come about?<br>This increase in temperature is an effect of progesterone. Progesterone is produced in the corpus luteum. The corpus luteum is formed after ovulation (follicle rupture) during the 2nd half of the cycle. (Ziegener, n.d.)<br>What happens to the body's core temperature once ovulation has taken place?<br>a) The core body temperature rises<br>b) The core body temperature drops<br>c) The core body temperature does not change due to ovulation (Trackle GmbH, n.d.)	When does the body temperature increase during the menstrual cycle?<br>- **After ovulation**<br>- At the start of menstruation<br>- At the end of menstruation<br>- The temperature does not change
MC_15		Circle all of the symptoms that you believe may accompany PMS.<br>a) irritability<br>b) Tension<br>c) headache<br>d) depression<br>e) fatigue<br>f) breast swelling<br>g) increased appetite<br>h) constipation<br>i) abdominalbloating<br>j) anxiety<br>k) forgetfulness<br>l) insomnia<br>m) cold sweats<br>n) dizziness<br>o) nausea<br>p) I am not sure (Johnson, 2008)	Which of the symptoms may be associated with premenstrual syndrome (PMS)?<br>- Irritability, tension, chest pain, dizziness, forgetfulness<br>- Headaches, tiredness, insomnia, emotional upsets<br>- Increased appetite, constipation, abdominal bloating, nausea<br>- **All of the above**

MC_16			Name the four phases of the menstrual cycle!	What are the two different phases of the menstrual cycle?<br>a) Proliferation phase<br>b) Secretion phase (Ziegener, n.d.)	What is the correct order of phases during the menstrual cycle?<br>– **Menstruation, follicle maturation, ovulation, luteal phase**<br>- Menstruation, luteal phase, ovulation, follicle maturation<br>- Luteal phase, menstruation, ovulation, follicle maturation<br>- Ovulation, follicle maturation, menstruation, luteal phase
MC_17	Where is the cycle controlled?	Which organs are involved in the hormonal regulation of the female cycle?	Where in the body does the formation and release of LH and FSH take place?	Name the two endocrine glands responsible for producing the hormones of the female menstrual cycle:<br>a) Pituitary gland<br>b) Ovary<br>c) Oestrogens<br>d) LH (Quizizz, n.d.)	Where is the menstrual cycle controlled?<br>- **Hypothalamus, pituitary gland (hypophysis), ovaries (ovaries)**<br>- Pituitary gland (hypophysis), ovaries (ovaries), vagina (vagina)<br>- Hypothalamus, ovaries (ovaries), pancreas (pancreas)<br>- Hypothalamus, pituitary gland (hypophysis), vagina (vagina)
MC_18			Which two hormones peak in concentration at the time of ovulation?	Which hormone triggers ovulation?<br>a) Oestrogen<br>b) Progesterone<br>c) LH<br>d) FSH (Krahlisch, 2013)<br>How does LH work?<br>a) Oocyte maturation<br>b) ovulation triggering<br>c) Structure of the endometrium<br>d) Breakdown of the uterine lining (Quizizz, n.d.)<br>Which hormone is responsible for release of dominant follicle from ovary into the oviduct?<br>a) FSH<br>b) Estrogen<br>c) LH<br>d) Progesterone (ProProfs Editorial Team, 2023)	Which hormone peak triggers ovulation?<br>- Oestrogen peak<br>- Progesterone peak<br>- **LH peak**<br>- FSH peak
MC_19				Which hormone is responsible for producing symptoms of PMS?<br>a) FSH<br>b) Estrogen<br>c) LH<br>d) Progesterone (ProProfs Editorial Team, 2023)	Which hormone is responsible for the symptoms of premenstrual syndrome (PMS)?<br>- FSH<br>- Oestrogen<br>- LH<br>- **Progesterone**

MC_20		Where is oestrogen found, and what is the function of this hormone?	What is the role of the hormone oestrogen in the pre-ovulatory phase?<br>a) Increase in cervical mucus production<br>b) Reduction of the endometrium<br>c) Prevention of renewed ovulation<br>d) Increase in basal body temperature (Krahlisch, 2013)<br>Describe the effect of the sex hormones oestrogens and gestagens in the female cycle:<br>a) Trigger ovulation<br>b) Breakdown of the uterine lining<br>c) Structure of the endometrium<br>d) Trigger egg maturation (Quizizz, n.d.)<br>Which of the following are effects of increased levels of oestrogen in the follicular phase of the menstrual cycle?<br>a) Hair thinning<br>b) Thickening of cervical mucous<br>c) Thinning of cervical mucous<br>d) Thickening of the endometrium (Potter, 2011)	Which of the following are the effects of increased oestrogen levels in the follicular phase of the menstrual cycle?<br>– Thinner hair<br>- Thickening of the cervical mucus<br>- Dilution of the cervical mucus<br>- **Thickening of the lining of the uterus**
MC_21	Which hormones are produced in the ovary?		Name the female ovarian hormones that fluctuate throughout the menstrual cycle: (Larsen et al., 2020)<br>Which hormones are produced in the ovary?<br>a) LH and FSH<br>b) Ocytocin and cortisol<br>c) Oestrogen and progesterone<br>d) Adrenaline and noradrenaline (Krahlisch, 2013)<br>Which hormones play a decisive role in the female menstrual cycle?<br>a) FSH, LH, oestrogen and progesterone<br>b) Adrenaline, FSH, insulin and cortisol<br>c) Testosterone, progesterone and oestrogen<br>d) Oxytocin, oestrogen, prolactin and testosterone (Trackle GmbH, n.d.)	Which statement is correct?<br>- **Oestrogen and progesterone are produced in the ovaries.**<br>- Oestrogen and LH are produced in the vagina.<br>- FSH and LH are produced in the pancreas.<br>- LH and progesterone are produced in the pituitary gland (hypophysis).

Notes: The correct answer is marked in bold. The answer format “Don't know” was also added to each item.

References

ABC Health & Wellbeing. (2017, October 5). Period quiz: Do you know menstruation fact from fiction? Retrieved from https://www.abc.net.au/news/health/2017-10-05/do-you-know-menstruation-fact-from-fiction/9010430. [Accessed on June 6, 2024]

Begum, J. (2023, August 29). Is my period normal? WebMD. Retrieved from https://www.webmd.com/women/cm/rm-quiz-period-normal. [Accessed on June 6, 2024]

Benshaul-Tolonen, A., Aguilar-Gomez, S., Heller Batzer, N., Cai, R., & Nyanza, E. C. (2020). Period teasing, stigma and knowledge: A survey of adolescent boys and girls in Northern Tanzania. *PLoS One, 15*(10), e0239914. https://doi.org/10.1371/journal.pone.0239914

Erdbeerwoche. (2017). Erste repräsentative Umfrage zum Wissensstand und zur Einstellung österreichischer Jugendlicher zur Menstruation und zur Monatshygiene [First representative survey on the knowledge and attitudes of Austrian teenagers towards menstruation and menstrual hygiene]. Retrieved from https://www.ready-for-red.at/umfrage-menstruation/

Harvey-Jenner, C., & Millington, H. (2023, August 23). How much do you really know about your period? Cosmopolitan. Retrieved from https://www.cosmopolitan.com/uk/body/health/a33069814/period-quiz/. [Accessed on June 6, 2024]

Johnson, T. R. (2008). *Knowledge and Attitudes Regarding the Menstrual Cycle, Oral Contraceptives, and Sport Performance: The Conceptualization and Development of a Questionnaire for Athletic Coaches* [Dissertation]. Florida State University Libraries, Florida.

Johnson & Johnson GmbH. (n.d.). Aufklärungsstunde [Awareness session]. Retrieved from https://www.aufklaerungsstunde.de/. [Accessed on June 6, 2024]

Krahlisch, M. (2013, July 1). Menstruationszyklus-Quiz - Teste Dich [Menstrual cycle quiz - test yourself]. Retrieved from https://www.testedich.de/gesundheitstests/verhuetung-kinderwunsch-schwangerschaft/periode-regel-tage/quiz32/1372064207/menstruationszyklus-quiz. [Accessed on June 6, 2024]

Larsen, B., Morris, K., Quinn, K., Osborne, M., & Minahan, C. (2020). Practice does not make perfect: A brief view of athletes' knowledge on the menstrual cycle and oral contraceptives. *Journal of Science and Medicine in Sport, 23*(8), 690–694. https://doi.org/10.1016/j.jsams.2020.02.003

Mädchen.de. (2021, February 12). Test: Wie gut kennst Du Dich mit der Periode aus? [Test: How well do you know your period?] Retrieved from https://www.maedchen.de/love/periode-menstruation-quiz-wissen. [Accessed on June 6, 2024]

Potter, L. (2011, June 28). Menstrual Cycle Quiz. Retrieved from geekymedics.com/menstrual-cycle-quiz. [Accessed on June 6, 2024]

ProProfs Editorial Team. (2023, March 22). Menstrual Cycle Quiz Questions And Answers [Approved & Edited by ProProfs Editorial Team | By Sana]. Retrieved from https://www.proprofs.com/quiz-school/story.php?title=menstrual-cycle_6ca. [Accessed on June 6, 2024]

Quizizz. (n.d.). Weiblicher Zyklus [Female menstrual cycle]. Retrieved from https://quizizz.com/admin/quiz/5cf6164b0ff2cc001a76e64b/weiblicher-zyklus. [Accessed on June 6, 2024]

Szűcs, M., Bitó, T., Csíkos, C., Párducz Szöllősi, A., Furau, C., Blidaru, I., Kapamadzija, A., Sedlecky, K., & Bártfai, G. (2017). Knowledge and attitudes of female university students on menstrual cycle and contraception. *Journal of Obstetrics and Gynaecology, 37*(2), 210-214. doi:10.1080/01443615.2016.1229279.

Trackle GmbH. (n.d). Zyklus Quiz [Menstrual Cycle quiz]. Retrieved from https://trackle.de/zyklus/zyklus-quiz/. [Accessed on June 6, 2024]

Your Fertility Journey. (2018). Menstrual cycle quiz. Retrieved from https://yourfertilityjourney.com/menstrual-cycle-quiz/. [Accessed on June 6, 2024]

Ziegener, G. (n.d.). Menstruation cycle: Basic knowledge. Retrieved from http://heilberufe-ausbildung.de/Geschlechtsorgane/Menstruation.htm. [Accessed on June 6, 2024]

Table S2 Relevance and clarity for each item in the 12-item Menstrual Cycle Knowledge Questionnaire (MCKQ), as rated by the expert panel (*n* = 18)

	Relevance					Clarity				
	M	SD	Median	min	max	M	SD	Median	min	max
MC_01	5.4	0.8	6	3	6	5.4	0.9	6	3	6
MC_04	5.3	0.9	6	3	6	5.6	0.8	6	3	6
MC_06	5.3	0.9	6	3	6	5.2	1.0	6	3	6
MC_07	5.6	0.7	6	4	6	5.7	0.6	6	4	6
MC_10	5.5	0.7	6	4	6	4.8	1.2	5	2	6
MC_11	5.2	0.8	5	4	6	5.1	1.0	5	2	6
MC_12	5.7	0.5	6	5	6	5.6	0.8	6	3	6
MC_14	5.2	1.0	6	3	6	5.3	1.2	6	2	6
MC_15	5.1	0.9	5	3	6	5.4	0.8	6	3	6
MC_16	5.5	0.7	6	4	6	5.7	0.6	6	4	6
MC_17	5.3	0.8	5	3	6	5.7	0.6	6	4	6
**Total**	**5.4**	**0.8**	**6**	**3**	**6**	**5.4**	**0.9**	**6**	**2**	**6**

Notes: M = mean value, SD = standard deviation, min = minimum, max = maximum. Relevance: 1 = not at all relevant, 6 = very relevant. Clarity: 1 = not at all clear, 6 = very clear.

Table S3 Item descriptions (English translations) for the final 12-item Menstrual Cycle Knowledge Questionnaire (MCKQ) including comments from the expert panel (*n* = 18) (survey, videoconference)

	Comments (Survey/Videoconference)	Item (English)
MC_01	Young women/girls	At what age, on average, do girls get their period (menarche) for the first time?<br>– 9-11 years of age<br>– **11-14 years of age**<br>– 15-17 years of age<br>– > 17 years of
MC_04	Re-formulate the answer option:<br>1st day of real bleeding	When is the first day of the menstrual cycle?<br>– The day ovulation occurs<br>– The last day of the period<br>– The first day after period<br>– **The first day of period**
MC_06	Re-formulate the answer option:<br>Function check? -> Functionality check of the uterus<br>Endometrium<br>Cleaning hormone balance -> Elimination of defective blood cells	What is the cause of bleeding?<br>– **Rejection of the "old" endometrium**<br>– Termination of pregnancy<br>– Functionality check of the uterus<br>– Elimination of defective blood cells
MC_07		If the egg has not been fertilised....<br>– then period starts about one week after ovulation.<br>– **then, the period starts about two weeks after ovulation.**<br>– then period starts about three weeks after ovulation.<br>– then period starts about four weeks after ovulation.
MC_10	What causes/by which mechanisms are reasonable for menstrual bleeding?<br>Re-formulate the answer options:<br>Drop in hormones? -> Drop in hormones leads to shedding of uterine lining<br>Uterine haemorrhage? -> Blood supply from the uterus (womb) is diverted	Which mechanisms are reasonable for menstrual bleeding?<br>– Blood supply from the uterus (womb) is diverted<br>– Micro-tears during ovulation<br>– **Drop in hormones leads to shedding of uterine lining**<br>– Hormone constellation increases blood production
MC_11	Other causes such as endometriosis must be considered<br>Re-formulate the answer option:<br>Psyche is associated with the development of pain -> Inflammatory reaction	What are the primary reasons for menstrual pain during bleeding?<br>– **Uterine contractions**<br>– Ovulation<br>– Opening of the cervix<br>– Inflammatory reaction
MC_12	If necessary, distinguish between primary and secondary amenorrhoea	What is amenorrhea?<br>– **Absence of period**<br>– intermenstrual bleeding<br>– Development of the ovaries<br>– Painful proliferation of the tissue of the endometrium

MC_14	"Increase in core body temperature over approx. 2 weeks" or to make it more clear What triggers the body's temperature increase during the menstrual cycle?<br>Depend on the question re-formulate the answer option:<br>– shortly after ovulation<br>– in the second half of the cycle, shortly after ovulation<br>– in the first half of the cycle, ...	What triggers the body's temperature increase during the menstrual cycle?<br>– **ovulation**<br>– the beginning of the period<br>– the end of period<br>– the basal body temperature does not change
MC_15	Additional possible answer option:<br>Tendency to oedema -> constipation. Flatulence. Water retention	Which are not accompanying symptoms of premenstrual syndrome (PMS)?<br>– irritability. emotional upset. Headache<br>– chest pain. fatigue. increased appetite<br>– constipation. flatulence. water retention<br>– **urine leakage. fever episodes. colour change of nipples**
MC_16	Correct order of phases<br>Re-formulate the answer option:<br>Luteal phase?	What is the correct order of phases during the menstrual cycle?<br>– bleeding. luteal phase. ovulation. follicle phase<br>– **bleeding. follicle phase. ovulation. luteal phase**<br>– Luteal phase. bleeding. ovulation. follicle phase<br>– ovulation. follicular phase. bleeding. luteal phase
MC_17	Where is…	Where is the menstrual cycle controlled?<br>– Pituitary gland (hypophysis). ovaries (ovaries). vagina (vagina)<br>– Hypothalamus. ovaries (ovaries). pancreas (pancreas)<br>– Hypothalamus. pituitary gland (hypophysis). vagina (vagina)<br>– **Hypothalamus. pituitary gland (hypophysis). ovaries (ovaries)**
MC_21		Which statement about hormones is false?<br>– Estrogen has a muscle-building (anabolic) effect.<br>– Progesterone has a muscle-degrading (catabolic) effect.<br>– **Testosterone is exclusively produced in the male body.**<br>– The increase in luteinising hormone (LH peak) triggers ovulation.

Notes: The correct answer is marked in bold. The answer format "Don't know" was added to each item.
